# Clinical effect of fast-track surgery on laparoscopic treatment of pediatric acute appendicitis and its effect on abdominal inflammation and stress level

**DOI:** 10.12669/pjms.41.7.11153

**Published:** 2025-07

**Authors:** Wen-long Liu, Shan-po Wei, Lin-feng Zou, Sheng-chuan Tian, Yan-yan Liu

**Affiliations:** 1 Wen-long Liu, Department of General Surgery, Baoding Hospital, Beijing Children’s Hospital Affiliated to Capital Medical University, Baoding, Hebei, 071000, P.R. China; 2 Shan-po Wei, Department of General Surgery, Baoding Hospital, Beijing Children’s Hospital Affiliated to Capital Medical University, Baoding, Hebei, 071000, P.R. China; 3 Lin-feng Zou, Department of General Surgery, Baoding Hospital, Beijing Children’s Hospital Affiliated to Capital Medical University, Baoding, Hebei, 071000, P.R. China; 4 Sheng-chuan Tian, Department of General Surgery, Baoding Hospital, Beijing Children’s Hospital Affiliated to Capital Medical University, Baoding, Hebei, 071000, P.R. China; 5 Yan-yan Liu, Department of General Surgery, Baoding Hospital, Beijing Children’s Hospital Affiliated to Capital Medical University, Baoding, Hebei, 071000, P.R. China

**Keywords:** Abdominal inflammation, Fast-track surgery, Laparoscopic appendectomy, Pediatric acute appendicitis, Stress

## Abstract

**Objective:**

To observe the clinical effect of fast-track surgery on laparoscopic treatment of pediatric acute appendicitis and its effect on abdominal inflammation and stress level.

**Methods:**

This was a clinical comparative study. Ninety children admitted to Baoding Hospital, Beijing Children’s Hospital Affiliated to Capital Medical University from July 2022 to December 2023 for laparoscopic appendectomy were randomly divided into the control group and the experimental group(n=45 in each group). The control cohort received routine perioperative nursing care, whereas the experimental cohort received fast-track surgery during the same perioperative period. Postoperative complications, including abdominal distension, nausea, vomiting, urinary tract infection, poor incision healing, were compared between the two cohorts. Evaluated the contrasts in stress biomarkers, including TNF α, IL-6, CRP, and serum cortisol (cort), as well as pain symptoms and satisfaction levels between the control and experimental cohorts.

**Results:**

The experimental cohort exhibited considerably shorter postoperative exhaust time, feeding time, getting out-of-bed time, and hospital stay compared to the control cohort, with statistically meaningful contrast(P=0.00). At 12 and 24 hours after surgery, the VAS scores in the experimental cohort were considerably lower than those in the control cohort, with a statistically meaningful contrast(P=0.00). Furthermore, the nursing satisfaction rate in the experimental cohort was considerably higher than in the control cohort, with a statistically meaningful contrast (P=0.02).

**Conclusion:**

Fast-track surgery boasts a variety of benefits in the perioperative period of laparoscopic treatment of pediatric acute appendicitis, such as effectively shortening postoperative recovery time and hospital stay, reducing the occurrence of complications.

## INTRODUCTION

Appendicitis, a common acute abdominal condition, tends to make inroads on people of any age, even in its acute state. Pediatric appendicitis is an infectious disease of the appendix^1^,^2^ which is commonly seen in pediatric surgery. It is characterized by strong destructiveness, rapid progression, as well as prone to gangrene and perforation^3^, posing a serious threat to the lives of children. Pediatric appendicitis has a low incidence in infants and toddlers, while it is mostly found in the preschool age cohort. Children are a common cohort for the onset of acute appendicitis, presenting severe pain and inducing symptoms such as hyperpyrexia and vomiting, while lacking typical abdominal signs.^4^ Given the difficulty of diagnosis and the risk of perforation, prompt diagnosis and early treatment of acute appendicitis are required.^5^

Currently, surgery is clinically the preferred mode of treatment for acute appendicitis, and due to the popularization and application of minimally invasive surgery, laparoscopic appendectomy boasts advantages such as less surgical trauma and shorter recovery time.^6^ However, minimally invasive laparoscopic appendectomy is an invasive treatment modality that makes it difficult to eliminate complications. Children are at high risk of postoperative infection and intestinal adhesion due to their imperfect immune function.^7^,^8^ Fast-track surgery is a modern surgical concept that takes advantage of multidisciplinary approaches to reduce pain and trauma in order to shorten treatment time.^9^ In this study, fast-track surgery was applied in the laparoscopic treatment of pediatric acute appendicitis, and certain clinical results were achieved.

## METHODS

This was a clinical comparative study. Ninety children admitted to Baoding Hospital, Beijing Children’s Hospital Affiliated to Capital Medical University from July 2022 to December 2023 for laparoscopic appendectomy were randomly divided into two groups: the control group and the experimental group, with 45 cases in each group. Data was collected through the hospital medical record system.

### Ethics approval

This research received ethical approval from the Institutional Ethics Committee of Baoding Hospital, Beijing Children’s Hospital Affiliated to Capital Medical University (No.:2023-27; Dated: May 12, 2023), and written informed consent was obtained from guardians of all participants prior to their inclusion in the study.

### Inclusion criteria:

- Children meeting the diagnostic criteria for acute appendicitis.^10^
- Children who met the indications for laparoscopic appendicitis surgery and opted for laparoscopic surgery for treatment.
- Children 6≤14 years old.
- Children with onset time ≤72 hours.
- Children with a combination of contraindications to surgery other than considerable organ disease.
- Children who had good treatment compliance and were able to understand and voluntarily cooperate with the completion of the study.
- The guardians of the children agreed to the study and signed the informed consent.
- Children with complete clinical data.

### Exclusion criteria:

- Children who were unable to tolerate surgery due to combined dysfunction of other vital organs such as the heart, liver and kidneys.
- Children with severe mental disorder or cognitive dysfunction.
- Children with perforated appendix and diffuse peritonitis.
- Children with combined abdominal diseases.
- Children with coagulation function and immune function deficiency.

All pediatric patients underwent laparoscopic appendectomy. Both groups received standardized treatment immediately after admission. The control cohort received routine nursing care during the perioperative period. The families were informed of the necessity of laparoscopic surgery and their full understanding and cooperation was obtained; The children were asked to fast for 10 hours and not to drink for 6-8 hours; in addition, close communication was maintained with the families to stabilize the children. The children were monitored at all times, kept warm during the operation, and received psychological and dietary nursing.

In contrast, children in the observation cohort received a fast-track surgery during the perioperative period. The details are as follows:

### Knowledge education:

In response to the children’s condition, knowledge about the disease and the importance of treatment was explained to the children’s family, and the precautions to be taken during treatment and possible complications after surgery were explained to them so that they could actively cooperate with the nursing staff and facilitate the children’s early recovery.

### Psychological nursing:

psychological counselling was provided to the children and their families after admission, so that the children could accept treatment voluntarily; the families were advised to communicate more actively with the children to eliminate their resistance and help the children build up their confidence in treatment.

### Preoperative preparation:

the children and their families were informed of the knowledge related to laparoscopic surgery, and the children were instructed to reasonably refrain from drinking and fasting before surgery. In addition, a comprehensive physical examination was carried out on the children, and the surgical instruments are strictly disinfected and cleaned to ensure the smooth implementation of the surgery and to avoid postoperative infections.

### Intraoperative nursing:

the changes of various indicators during the surgery of children were closely monitored. If there were any abnormalities, detailed records were recorded and symptomatic treatment was performed. At the same time, infusion treatment and pain management were carried out to record the physical condition of the children, and the intervention plan was adjusted according to the actual situation of the children.

### Postoperative nursing:

after the surgery, the children’s daily recovery was truthfully recorded, daily infusion therapy was carried out, and treatment measures were given according to medical advice; The children were instructed to eat a reasonable diet, avoiding spicy and irritating foods, and to eat as light a diet as possible, with more nutritious and high-protein foods to ensure balanced nutrition; Moreover, the children’s daily bowel movements were recorded and nursing was tailored to prevent post-operative complications.

### Observation indexes:

- Comparative analysis of clinical outcomes: the contrasts in postoperative exhaust time, feeding time, getting out-of-bed time and hospital stay were compared between the two cohorts;
- Comparison of postoperative complications, such as abdominal distension, nausea, vomiting, urinary tract infection, and poor incision healing, between the two cohorts.
- Comparative analysis of inflammatory and stress factor levels, which involved measuring TNF α, IL-6, CRP, and serum cortisol (cort) levels before and after the intervention through venous blood sampling.
- Comparative analysis of postoperative pain symptom scores, which were evaluated using the visual analogue scale (VAS) at 1 hour, 12 hours, and 24 hours after surgery. The scale ranged from 0 to 10, with higher scores indicating more severe pain.^11^
- The Patient Satisfaction Questionnaire Short Form (PSQ-18)^12^ was used to compare the satisfaction levels of the pediatric patients before and after the intervention. The survey contained answers including extremely content, moderately content, content, indeterminate, and discontent. Overall contentment was computed by taking the sum of extremely content, moderately content, and content responses, divided by the total number of cases and multiplied by 100%.

### Statistical analysis:

All statistical analyses were performed using SPSS20.0 software, and the results of the measurement data were presented as (*χ̅*±*S*). The independent sample t-test was used to compare the two cohorts, while the paired t-test or analysis of variance was used for intra-cohort data analysis. Additionally, the χ^2^ test was applied for comparing rates, with a P-value <0.05 indicating statistical significance.

## RESULTS

In the experimental group, there were 26 males and 19 females, aged 5-13 years, with a mean of 9.45±2.47 years, while in the control group, there were 28 males and 17 females, aged 5-12 years, with a mean of 9.16±2.76 years. No significant differences were observed between the two groups in the comparison of general data (P>0.05), indicating the comparability of differences between the groups (Table-I).

**Table-I:** Comparative analysis of general data between the two cohorts (*χ̄*±*S*) n=45.

Index	Experimental cohort	Control cohort	t/χ^2^	p
Male (cases, %)	26 (58%)	28 (62%)	0.19	0.67
Age (years)	9.45±2.47	9.16±2.76	0.54	0.59
Body weight (kg)	30.36±3.08	31.04±3.17	1.03	0.31
Type of appendicitis			0.18	0.67
Simple	21 (47%)	23 (51%)		
Suppurative	24 (53%)	22 (49%)		
Course of disease (d)	3.06±1.05	3.13±1.21	0.33	0.75

P>0.05.

Statistically meaningful contrasts (P=0.00) were observed between the experimental and control cohorts regarding postoperative exhaust time, feeding time, getting out-of-bed time, and hospital stay, with the experimental cohort exhibiting considerably shorter durations than the control cohort (Table-II).

**Table-II:** Comparative analysis of clinical outcomes between the two cohorts (*χ̄*±*S*) n=45.

Group	Exhaust time (d)*	Feeding time (h)*	Getting out-of-bed time (h)*	Hospitalization time (d)*
Experimental cohort	20.33±5.28	9.36±1.25	7.84±1.54	5.87±1.25
Control cohort	24.67±5.72	16.87±5.24	12.62±3.08	8.76±2.13
*t*	3.73	9.36	9.32	7.83
*p*	0.00	0.00	0.00	0.00

* P<0.05.

Postoperative complications in both cohorts were abdominal distension, nausea, vomiting, urinary tract infection and poor wound healing. A statistically meaningful contrast (P=0.04) was found between the experimental and control cohorts in terms of the complication rate, with the experimental cohort exhibiting a lower rate of 7% compared to the control cohort’s rate of 22% (Table-III).

**Table-III:** Comparative analysis of postoperative complications between the two cohorts (*χ̄*±*S*) n=45

Group	Abdominal distension	Nausea	Vomiting	Urinary tract infection	Poor wound healing	Incidence*
Experimental cohort	1	1	1	0	0	3(7%)
Control cohort	6	1	2	1	0	10(22%)
*χ^2^*						4.41
*p*						0.04

* P<0.05.

**Table-IV:** Comparative analysis of the changes of inflammation and stress factors in the two cohorts before and after the intervention (*χ̄*±*S*) n=45.

Index		Experimental cohort	Control cohort	t	P
TNF-ɑ (ng/L)	Before intervention	35.76±8.23	34.81±8.72	0.53	0.60
	After intervention*	21.40±6.26	25.62±6.41	3.16	0.00
CRP (mg/L)	Before intervention	53.38±9.72	52.82±8.67	0.29	0.77
	After intervention*	23.71±6.35	26.72±6.07	2.30	0.02
IL-6 (ng/L)	Before intervention	18.27±3.92	17.80±3.54	0.60	0.56
	After intervention*	7.63±1.26	10.47±2.15	7.65	0.00
Cort (μg/L)	Before intervention	126.87±23.66	124.73±21.08	0.45	0.65
	After intervention*	52.72±13.07	58.80±10.15	2.46	0.00

* P<0.05.

Before the intervention, there was no statistically meaningful contrast (P>0.05) in the levels of inflammatory and stress factors such as TNF-a, IL-6, CRP, and Cort between the two cohorts, as they were meaningfully increased. However, after the intervention, the experimental cohort exhibited considerably lower levels of these factors compared to the control cohort, with a statistically considerable contrast (P<0.05).

There was no statistically meaningful contrast (P=0.75) in the VAS scores of the two cohorts one hour after surgery. However, at 12 hours and 24 hours after operation, the experimental cohort exhibited considerably lower VAS scores compared to the control cohort, with a statistically considerable contrast (P=0.00) (Table-V). The experimental cohort achieved a considerably higher nursing satisfaction rate of 100% compared to the control cohort’s rate of 89%, with a statistically considerable contrast (P=0.02) (Table-VI).

**Table-V:** Comparative analysis of postoperative pain symptom scores between the two cohorts (*χ̄*±*S*) n=45.

Group	1 hour after surgery	12 hours after surgery*	24 hours after surgery*
Experimental cohort	5.44±1.36	4.07±0.33	2.27±0.45
Control cohort	5.36±1.32	4.67±0.48	2.64±0.48
t	0.32	6.94	3.85
p	0.75	0.00	0.00

* P<0.05.

**Table-VI:** Comparative analysis of satisfaction between the two cohorts (*χ̄*±*S*) n=45.

Group	Very content	Relatively content	Content	Uncertain	Dissatisfied	Total satisfaction*
Experimental cohort	40	3	2	0	0	45 (100%)
Control cohort	27	9	4	2	3	40 (89%)
*χ^2^*						5.29
*P*						0.02

* P<0.05.

## DISCUSSION

Our study found that the experimental cohort had considerably shorter postoperative exhaust time, feeding time, time to get out of bed, and hospital stay compared to the control cohort, with statistically considerable contrasts (P=0.00). This may be due to the fact that fast-track surgery adheres to the concept of “patient-centered” and implements quality perioperative nursing that takes into account the contrasts of patients, thus speeding up the recovery of the body, effectively shortening the recovery time and improving the children’s experience and satisfaction with nursing. The results of our study confirmed that nursing satisfaction in the experimental cohort was 100%, which was dramatically greater than the 89% observed in the control cohort, with a statistically meaningful contrast (P=0.02). In a comparative study, Arena et al.^13^ demonstrated that fast-track surgery can effectively restore gastrointestinal function and reduce postoperative complications in patients undergoing gastrointestinal surgery.

In current clinical practice, conventional nursing is mostly used in the perioperative period for children with acute appendicitis. Conventional nursing is the common mode of nursing for patients with acute appendicitis in the perioperative period, focusing only on disease nursing while paying less attention to patients in the nursing process, resulting in unsatisfactory nursing effects. With the transformation and upgrading of the clinical medical model, China’s nursing concept is also in a rapid development stage. Fast-track surgery has been gradually applied in the perioperative period of surgical operation, which has improved the surgical outcomes.^14^

Fast-track surgery is a concept promoted and implemented by surgeon Rove KO in recent years.^15^ It involves the adoption of a set of evidence-based perioperative optimization measures aimed at reducing or minimizing patients’ physical and psychological stress and achieving speedy recovery.^16^ When performing fast-track surgery, surgeons need to cooperate with anaesthetists, nursing staff, dietitians and family members to improve the management of patients and improve the quality and efficiency of nursing. Up to now, a number of applied studies have been conducted by scholars targeting fast-track surgery in the field of pediatric surgery.^17^ Compared to conventional nursing in the perioperative period, fast-track surgery is evidence-based and patient- and disease-focused, aiming at improving patient prognosis and speed recovery through a range of measures.

Surgery is an invasive operation that may cause different degrees of injury to the body and increase the inflammation and stress response of patients.^18^,^19^ IL-6, CRP, TNP-a and Cort, as common inflammatory and stress factors in clinical practice, serve as important markers to measure the inflammatory stress response of the body and the stability of the disease.^20^ If inflammatory stress occurs in the body, L-6, CRP, TNF-a, and Cort levels increase substantially and decrease when these reactions are effectively controlled. Do-Wyeld et al.^21^ concluded that fast-track surgery reduces inflammatory factors and restores gastrointestinal function in patients with hand-wood. This is consistent with the results of the present study, suggesting the benefits of fast-track surgery in reducing the inflammatory stress response and improving the prognosis of patients. There are several possible reasons for this.

Preoperatively, introducing fast-track surgery and related knowledge of surgery can improve patients’ understanding of the disease and surgical procedures, thus reducing their psychological stress response to a certain extent. Intraoperatively, keeping the children warm can reduce the irritation caused by fluids and reduce the inflammatory stress response of patients. Postoperatively, patients are encouraged to carry out rehabilitation training as soon as possible, guided to carry out active and passive limb training, and given antibiotics, which can prevent the occurrence of incision infection and reduce the inflammatory stress response of the body to a certain extent.^22^

The results of this research demonstrate that following the intervention, the experimental cohort had notably lower levels of inflammatory and stress biomarkers, including TNF-a, CRP, IL-6, and Cort, compared to the control cohort, with a statistically considerable contrast (P=0.00). Moreover, at both 12 hours and 24 hours post-surgery, the experimental cohort reported considerably lower pain levels, as measured by VAS scores, compared to the control cohort, with a statistically considerable contrast (P=0.00). The reason for this may be that giving patients multi-modality such as chatting and playing music after surgery can divert their attention, reduce their pain and inflammatory stress response, shorten the recovery time of white blood cells and ameliorate their prognosis.

### Limitations:

Nevertheless, shortcomings are still visible in this study: it was a single-center study with certain selection bias, and a small number of cases were included with a short follow-up period. In response to this, more multi-center and large sample cases need to be included in standardized clinical studies in the future, with a view to further objectively evaluating the pros and cons of this surgical approach.

## CONCLUSIONS

Fast-track surgery is effective in the perioperative period of laparoscopic treatment of pediatric acute appendicitis, boasting optimization of clinical indicators, reduction of inflammation, stress response, effective reduction of hospital stays, and alleviation of patients’ pain and postoperative complications, which is worthy of promotion and application.

### Authors’ Contributions:

**WL and** **SW:** Carried out the studies, participated in collecting data, and drafted the manuscript, and are responsible and accountable for the accuracy or integrity of the work.

**LZ and ST:** Performed the statistical analysis and participated in its design

**YL:** Participated in acquisition, analysis, or interpretation of data and draft the manuscript.

All authors have read and approved the final manuscript.
